# Pixel-wise assessment of cardiovascular magnetic resonance first-pass perfusion using a cardiac phantom mimicking transmural myocardial perfusion gradients

**DOI:** 10.1002/mrm.28296

**Published:** 2020-05-19

**Authors:** Xenios Milidonis, Muhummad Sohaib Nazir, Torben Schneider, Myles Capstick, Sita Drost, Gertjan Kok, Nikola Pelevic, Christian Poelma, Tobias Schaeffter, Amedeo Chiribiri

**Affiliations:** 1 School of Biomedical Engineering & Imaging Sciences, King’s College London, London, United Kingdom; 2 Philips Healthcare, Guilford, United Kingdom; 3 ZMT Zurich MedTech AG, Zurich, Switzerland; 4 Laboratory for Aero- and Hydrodynamics, Technische Universiteit Delft, Delft, Netherlands; 5 VSL B.V., Delft, Netherlands; 6 Physikalisch-Technische Bundesanstalt, Berlin, Germany

**Keywords:** cardiovascular magnetic resonance, myocardial blood flow, myocardial perfusion, phantom, quality assurance, transmural gradients

## Abstract

**Purpose:**

Cardiovascular magnetic resonance first-pass perfusion for the pixel-wise detection of coronary artery disease is rapidly becoming the clinical standard, yet no widely available method exists for its assessment and validation. This study introduces a novel phantom capable of generating spatially dependent flow values to enable assessment of new perfusion imaging methods at the pixel level.

**Methods:**

A synthetic multicapillary myocardial phantom mimicking transmural myocardial perfusion gradients was designed and manufactured with high-precision 3D printing. The phantom was used in a stationary flow setup providing reference myocardial perfusion rates and was scanned on a 3T system. Repeated first-pass perfusion MRI for physiological perfusion rates between 1 and 4 mL/g/min was performed using a clinical dual-sequence technique. Fermi function-constrained deconvolution was used to estimate pixel-wise perfusion rate maps. Phase contrast (PC)-MRI was used to obtain velocity measurements that were converted to perfusion rates for validation of reference values and cross-method comparison. The accuracy of pixel-wise maps was assessed against simulated reference maps.

**Results:**

PC-MRI indicated excellent reproducibility in perfusion rate (coefficient of variation [CoV] 2.4-3.5%) and correlation with reference values (R^2^ = 0.985) across the full physiological range. Similar results were found for first-pass perfusion MRI (CoV 3.7-6.2%, R^2^ = 0.987). Pixel-wise maps indicated a transmural perfusion difference of 28.8-33.7% for PC-MRI and 23.8-37.7% for first-pass perfusion, matching the reference values (30.2-31.4%).

**Conclusion:**

The unique transmural perfusion pattern in the phantom allows effective pixel-wise assessment of first-pass perfusion acquisition protocols and quantification algorithms before their introduction into routine clinical use.

## 1 Introduction

Recent advances in cardiovascular magnetic resonance (CMR) for first-pass myocardial perfusion have led to its recommendation for use in patients with intermediate pretest probability of significant coronary artery disease (CAD) by European guidelines.^1^ One of the most promising techniques is quantitative analysis of perfusion data for the production of pixel-wise perfusion maps,^2–5^ with emerging studies suggesting an accuracy for the detection of CAD comparable or superior to visual assessment by expert operators.^6–8^ In particular, pixel-wise quantification would allow the assessment of regional and local perfusion variations. It is known since the early 1970s that myocardial blood flow is characterized by a transmural (radial) variation, when Downey and colleagues measured the uptake of radioactive tracers in different transmural layers of the dog’s myocardium and observed a 30% decrease in the uptake from the subendocardium to the subepicardium during diastole.^9,10^ Such observations were recently confirmed with CMR first-pass perfusion.^11–13^

The accuracy and reproducibility of pixel-wise quantification methods have yet to be systematically evaluated under identical conditions. This is partially attributed to the lack of a clearly defined and transferable gold standard. Validation with the use of microspheres in large animals is the ex vivo reference.^14^ However, this method requires dedicated equipment and expertise that are not readily available and may have a limited accuracy for transmural perfusion measurements due to microsphere skimming.^15,16^ Positron emission tomography (PET) perfusion is currently the clinical reference but uses ionizing radiation, is expensive, and is not widely available.^17^ True cross-validation requires hybrid PET-MR machines for simultaneous imaging of patients under the same physiological and hemodynamic conditions.^17–19^

Ideally, a validation technique should be simple to use, accurate, and reproducible and require no cohorts of patients or animal sacrifice. To this end, Driscoll and colleagues proposed a phantom simulating two-compartmental exchange for dynamic contrast-enhanced (DCE) imaging.^20^ The phantom was shown to generate reproducible signal intensity-time (SI) curves during first-pass perfusion experiments with computed tomography (CT), PET, and single-photon emission CT and is currently the only commercially available phantom of its kind.^20,21^ Nevertheless, the phantom does not include a full heart model, generates a homogeneous flow distribution within the myocardium, and has not yet been used with MRI. Chiribiri et al developed a phantom with a four-chamber heart and a more complex myocardium.^22^ The myocardium consists of parallel tubes to simulate contrast dispersion across the capillary bed and has been used with MRI, PET and CT.^5,23,24^ However, the phantom lacks diversity in capillary size and its low manufacturing re-producibility potentially hampers its commercialization. Both aforementioned phantoms have proven useful in the assessment of perfusion methodologies, but their design limits their use to the measurement of spatially homogeneous perfusion rate and, therefore, allow only global perfusion validation.^5,21,23–25^ Others proposed the use of a real perfused heart in a hardware phantom for physiological perfusion experiments, though the sacrifice of large animals, its high costs, and complicated pre-scan preparations preclude routine use in the clinic.^15,26^

Perfusion phantoms offer distinct advantages in terms of reproducibility in generating DCE-MRI data and providing reliable reference values and, as such, have the possibility to be established as true gold standards across institutions. In this study, we propose a novel cardiac phantom with a synthetic myocardial component encompassing a diversity in capillary size and length to create a transmural gradient in perfusion rate of 30%, matching published in vivo measurements.^9,10^ A phantom setup yielding reference flow rates was also developed for imaging experiments, which is an upgraded version of a system previously described.^22^ The phantom was used to evaluate a standard clinical DCE-MRI protocol for pixel-wise quantification of first-pass perfusion over a range of physiological perfusion rates. In addition, phase contrast (PC)-MRI was used to validate global and transmural perfusion and perform cross-method comparison.

## 2 Methods

For clarity and consistency of the text, hereafter, the term “myocardium” refers to the developed synthetic myocardial component and the term “phantom” refers to the whole phantom setup including the myocardium. Each part of the phantom is referred to by the name of the anatomical structure it represents. Flow rate refers to the volumetric flow rate measured in units of [mL/min], and perfusion rate refers to the flow rate normalized to the tissue mass measured in units of [mL/g/min]. The tissue mass and tissue volume are often used interchangeably in myocardial perfusion quantification as the myocardial density is ~1 g/mL, and both refer to the contrast dispersion volume (e.g., the vascular space when intravascular contrast agents are used).^27^

### 2.1 Synthetic myocardium

**Figure 1:**
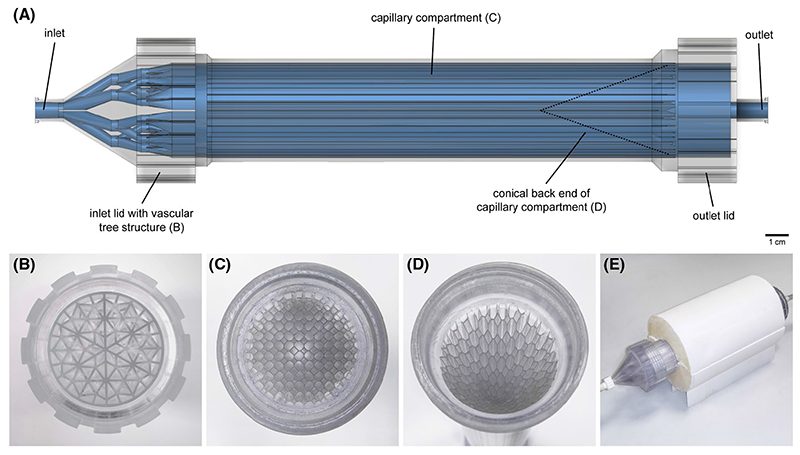
The geometry of the synthetic myocardium. Image (A) is a 3D rendering of the fully assembled synthetic myocardium showing the printed parts (transparent grey) and the internal contrast dispersion volume (blue). Photos show the inlet lid with the vascular tree structure (B), the front end of the capillary compartment (C), and the back end of the capillary compartment with the conical taper applying a 30% transmural difference in capillary length (D). The myocardium is placed on a water-filled cylindrical base providing signal for MRI data normalization (E)

**Figure 2:**
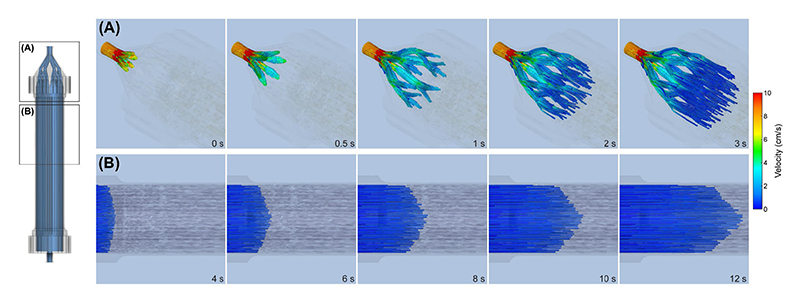
Computational fluid dynamics in the synthetic myocardium. The top row (A) shows the flow through the various branches of the vascular tree in the inlet lid over time. The bottom row (B) demonstrates the spatially dependent flow in the capillary compartment due to the transmural decrease in capillary length toward the center. Simulations were performed for a flow rate of 200 mL/min using approximately 500 particle traces. The times indicated in each frame are relative to the top-left instance

The myocardium consists of an inlet lid, a capillary compartment, and an outlet lid (Figure 1). The inlet lid was designed to resemble a vascular tree and splits a main coronary artery (inner diameter 7 mm) into 90 arterioles with fixed area (8.5 mm^2^) and variable cross-sectional shapes after three branching iterations (Figures 1 and 2). The path from the coronary artery to each arteriole has the same volume and length to evenly minimize the transport energy and ensure uniform distribution of the contrast agent in the arterioles. The inlet lid is succeeded by the capillary compartment, which is a cylindrical system of 229 capillaries with five different cross-sectional areas (1-6.4 mm^2^). At its tail end, this compartment has a conical taper that causes a 30% linear decrease in capillary length, radially from the outer wall to the central axis of the cylinder (Figures 1 and 2). This decrease results in a transmural decrease in flow resistance and, consequently, a 30% transmural increase in flow rate toward the center of the myocardium (see Supporting Information Table S1 for a detailed mathematical assessment of flow in the myocardium). The cylindrical shell of the capillary compartment has an inner diameter of 40 mm and a length up to the conical taper of 140 mm; this corresponds to a range of possible contrast dispersion volumes between 50 and 180 mL. The last part of the myocardium is the outlet lid consisting of a single coronary vein (inner diameter 7 mm). All myocardial parts can be disconnected for quality control, cleaning, and storage between imaging experiments.

The geometry of the myocardium was optimized based on initial measurements with PC-MRI and simulations using computational flow dynamics (CFD) to elicit physiological transit times for a contrast bolus traversing the capillaries (Figure 2). Flow simulations were performed in Autodesk^®^ CFD (version 17.2, San Rafael, California). The myocardium was printed with a transparent thermoplastic compound (Accura^®^ 60, 3D Systems^®^, Rock Hill, South Carolina) using a stereolithography machine with an accuracy 0.025-0.05 mm per 25.4 mm (ProX^®^ 800, 3D Systems).

### 2.2 Phantom setup

**Figure 3:**
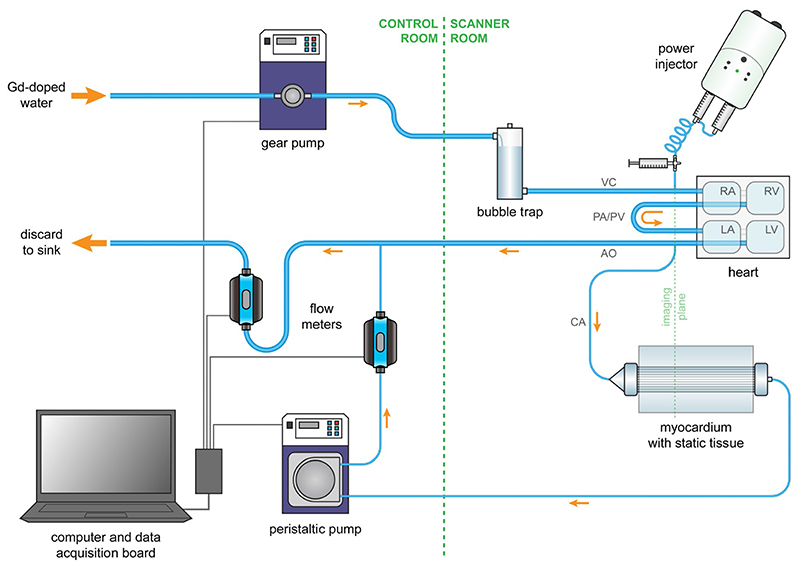
Schematic diagram of the phantom setup. A gear pump generates a constant flow of water across the phantom. Contrast agent is injected in the inferior vena cava using a power injector. Water and contrast agent flow into a four-chamber heart and exit via the aorta, which branches off to a coronary artery that supplies the synthetic myocardium. A control system located outside the scanner room consists of vertically mounted ultrasonic flow meters and a data acquisition board connected to a computer to monitor and adjust the cardiac output and myocardial flow rate. The typical imaging plane covers the aorta and myocardial capillary compartment where the arterial input function and myocardial signal intensity-time curves are sampled respectively. AO indicates aorta; CA, coronary artery; LA, left atrium; LV, left ventricle; PA, pulmonary artery; PV, pulmonary vein; RA, right atrium; RV, right ventricle; VC, vena cava

The myocardium was used for MRI in a setup comprising a cardiac phantom placed within the scanner and a control unit placed next to the scanner console (Figure 3). The control unit consists of a gear pump (MCP-Z, Ismatec^®^, Cole-Parmer GmbH, Wertheim, Germany) that constantly supplies the phantom with water. The phantom is made of a bubble trap that collects any air bubbles introduced in the hose from the water mains or during assembly. Water is then directed via the vena cava into the right atrium of a four-chamber heart made with machined acrylic. The heart contains two ventricular and two atrial chambers with volumes 129 mL and 106 mL respectively, corresponding to a 60 kg human subject. After exiting the left ventricle via the aorta, the water is driven from the phantom toward the control unit outside the scanner. The proximal aorta branches off to a coronary artery connected to the inlet of the myocardium, and the myocardial outlet is connected back to the control unit via a coronary vein (both inner diameter 6.5 mm). The myocardium is placed on a water-filled 3D printed cylindrical base that surrounds the capillary compartment (Figure 1). The base acts as a static tissue and provides a signal for coil sensitivity correction or normalization of PC-MRI data for eddy current-induced velocity offsets. For DCE-MRI, contrast agent is injected in the vena cava via a three-way tap and enters the cardiac and coronary circulation before exiting the system with the traversing water (Figure 3). All components of the cardiac phantom placed within the magnet are made with fully MR-compatible materials.

The outflow rate from the myocardium is monitored by an ultrasonic flow meter (Atrato 710-V10-D, Titan Enterprises, Sherborne, UK) and regulated by a peristaltic pump (MCP, Ismatec). The total cardiac output via the aorta and coronary vein passes through an additional flow meter (Atrato 740-V10-D), which is paired with the main gear pump for independent control. Plastic hoses with low volumetric expansion are used for the great vessels and the coronaries (inner diameter 10 mm and 6.5 mm respectively) and are attached to the various system components with standard plastic hose connectors or Luer fittings. The pumps and flow meters are connected to a data acquisition board that allows remote control of the phantom on a personal computer via a dedicated user interface developed in LabVIEW™ (National Instruments, Austin, Texas). The flow meters are calibrated by the manufacturer to an uncertainty of ±1% for measurements of cardiac outputs up to 5 L/min and myocardial flows up to 500 mL/min.

**Figure 4:**
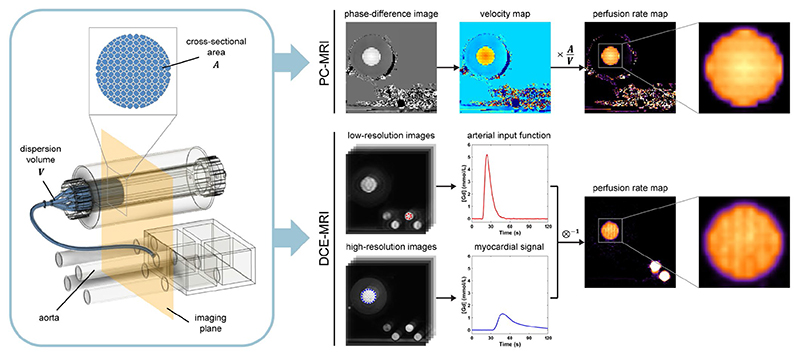
Overview of perfusion rate measurement from MRI data. Velocity maps from phase contrast (PC)-MRI are converted to perfusion rate maps by multiplication with the known total capillary cross-sectional area and division by the dispersion volume between the aorta and myocardium, including the coronary artery (blue). The same principle applies to conversion of reference myocardial flow rates set on the control system to ground truth perfusion rates, as well as conversion of simulated velocity maps by computational fluid dynamics. Perfusion rate from dynamic contrast-enhanced (DCE)-MRI images is estimated using standard deconvolution analysis of the arterial input and myocardial signals, obtained from the low- and high-resolution images respectively

Reference myocardial flow rates can be converted to ground truth velocities or perfusion rates using the known values of the capillary cross-sectional area at the imaging plane and the total myocardial dispersion volume, for direct comparison of flow and perfusion imaging methods (Figure 4).^28^ The system can be configured as a closed circuit by connecting the main input and output hoses. Because the aim of this study was to assess the reproducibility of CMR first-pass perfusion, the system was set up as an open circuit where the returning contrast-contaminated water is discarded to avoid build-up in baseline concentration (Figure 3). Before connection to the coronary vessels, the myocardium was primed with an aqueous solution of a nonionic surfactant (Tween^®^ 20, Merck, Darmstadt, Germany) in water at a 1:1000 dilution.

### 2.3 Image acquisition

Scanning was performed on a 3T system (Achieva, Philips Healthcare, Best, The Netherlands) equipped with a 32-channel cardiac phased-array coil. The phantom was supplied with water doped with Gadobutrol (Gadovist^®^, Bayer AG, Leverkusen, Germany) at 0.1 mmol/L to achieve clinical baseline *T*_1_ values in the blood pool at 3T. The optimal contrast dose was determined by preparing a set of eight Falcon tubes with various concentrations of the contrast agent between 0 and 10 mmol/L and measuring *T*_1_ using the MOLLI sequence (sampling scheme 5(3)3, 8 inversion times in the range 108-4108 ms, pulse repetition time (TR) 2.1 ms, echo time (TE) 0.9 ms, flip angle 20°, pixel bandwidth 1086 Hz; Supporting Information Figure S1).

The myocardial flow transmurality was validated using 2D gradient echo PC-MRI (10 acquisitions, temporal resolution 100 ms, velocity encoding [VENC] 3 cm/s, TR 10.9 ms, TE 8.7 ms, flip angle 10°, 4 averages, pixel bandwidth 383 Hz, field of view 256 × 256 mm^2^, resolution 2 × 2 mm^2^, slice thickness 8 mm). Myocardial perfusion was evaluated with an electrocardiogram-triggered single-shot saturation recovery spoiled gradient echo (SPGR) dual-sequence implementation used in our institution for clinical DCE-MRI (simulated heart rate 60 bpm, TR 2.3 ms, TE 1.2 ms, saturation recovery time 100 ms, flip angle 15°, pixel bandwidth 1085 Hz, field of view 256 × 256 mm^2^, slice thickness 8 mm, SENSE with acceleration factor 2).^29^ One myocardial high-resolution slice was acquired, placed at the same imaging plane as the low-resolution arterial input slice through the large thoracic vessels and the myocardial capillary compartment (Figure 3). The myocardial and arterial input slices were acquired at a resolution of 2.6 × 2.6 mm^2^ and 2.6 × 5.3 mm^2^ respectively and were reconstructed to 2 × 2 mm^2^ to enable direct comparison with PC-MRI data. Assuming a body mass of 60 kg, a single-bolus of 0.05 mmol/kg Gadobutrol was injected in the vena cava at 4 mL/s using an injector pump (Spectris Solaris, Medrad^®^, Bayer AG), followed by 30 mL of saline flush, according to contemporary recommendations for clinical first-pass perfusion imaging.^30^ The contrast bolus was injected 8 seconds after the start of the acquisition to provide sufficient baseline signal and each scan lasted 3 minutes to capture the full wash out of the contrast agent from the myocardium. To allow conversion of SI to gadolinium concentration, a pre-contrast *T*_1_ map was obtained prior to acquisition of DCE-MRI data using the MOLLI sequence (sampling scheme 5(3)3, 8 inversion times in the range 157-3157 ms, TR 3.6 ms, TE 1.4 ms, flip angle 15°, pixel bandwidth 393 Hz, field of view 256 × 256 mm^2^, resolution 2 × 2 mm^2^, slice thickness 8 mm). Imaging with each sequence was repeated five times in an interleaved fashion for each of four different reference myocardial flow rates (100, 200, 300, and 400 mL/min). The total contrast dispersion volume from the arterial input sampling location in the low-resolution slice to the high-resolution myocardial slice was 100 mL; therefore, the reference mean perfusion rates ranged between 1 and 4 mL/g/min (assuming a water density of 1 g/mL). An average of 10 minutes delay between repeats was used to ensure complete clearance of the contrast agent from the phantom. The phantom’s cardiac output was fixed at 3 L/min.

### 2.4 Image analysis

PC-MRI data were analyzed to generate myocardial pixelwise velocity maps. All 10 phase-difference images per scan were converted to velocity maps using the set VENC parameter. Eddy current-induced velocity offsets were corrected by fitting a quadratic polynomial surface to the static tissue signal surrounding the myocardium and subtracting the surface from velocity maps. The 10 velocity maps per scan were averaged to obtain a single map for assessment.

For pixel-wise perfusion quantification of DCE-MRI data, the arterial input function (AIF) was sampled in a circular region of interest placed in the aorta (diameter 10 mm). Both the AIF and myocardial SI curves were converted to gadolinium concentration using the precontrast *T*_1_ values and the signal model for SPGR, as previously described.^31^ Signal saturation and scaling differences were further alleviated by scaling the AIF so that its time-integral (corresponding to the contrast dispersion volume) matched that of the mean myocardial curve.^3,32^ Pixel-wise perfusion rate was then estimated using Fermi function-constrained deconvolution.^33^ Figure 4 provides an overview of perfusion rate measurement using each MRI method.

High-resolution reference velocity maps were obtained using CFD simulations for all four reference perfusion rates (Supporting Information Figure S2). Velocity maps from both PC-MRI and simulations were converted to perfusion rate maps by multiplication with the known total capillary cross-sectional area and division by the dispersion volume. Mean perfusion rate for PC-MRI and DCE-MRI maps was measured over the whole myocardial cross-section. To evaluate the maps at the pixel level, they were filtered using a Wiener filter, spatially interpolated to the resolution of reference maps and then aligned with the reference maps using registration by translation. Accuracy and repeatability were assessed using pixel-wise root mean square error (RMSE) and percentage coefficient of variation (CoV) maps. The transmural variation in perfusion rate was examined by sampling the maps across line profiles radially from the center of the myocardium to the outer boundary. Relative measurements of transmural variation were also obtained by normalization to the value in the center. Automated or semiautomated MATLAB routines developed in house were used for all analyses (version 2018b; MathWorks^®^, Natick, Massachusetts).

### 2.5 Statistical analysis

For the two MRI methods, the mean and pixel-wise RMSE and CoV were estimated from original and post-processed perfusion maps respectively. Linear regression analysis was used to assess the relationship between PC-MRI and DCE-MRI-based mean perfusion rates and reference values. The agreement between methods was examined using Bland-Altman analysis. One-way repeated measures analysis of variance with post hoc pairwise comparisons with Bonferroni correction was used to compare the mean perfusion rate. A *P* value less than 0.05 was considered statistically significant. Statistical analysis was performed in MATLAB and SPSS^®^ (version 25.0; IBM Corp., Armonk, New York).

## 3 Results

**Figure 5:**
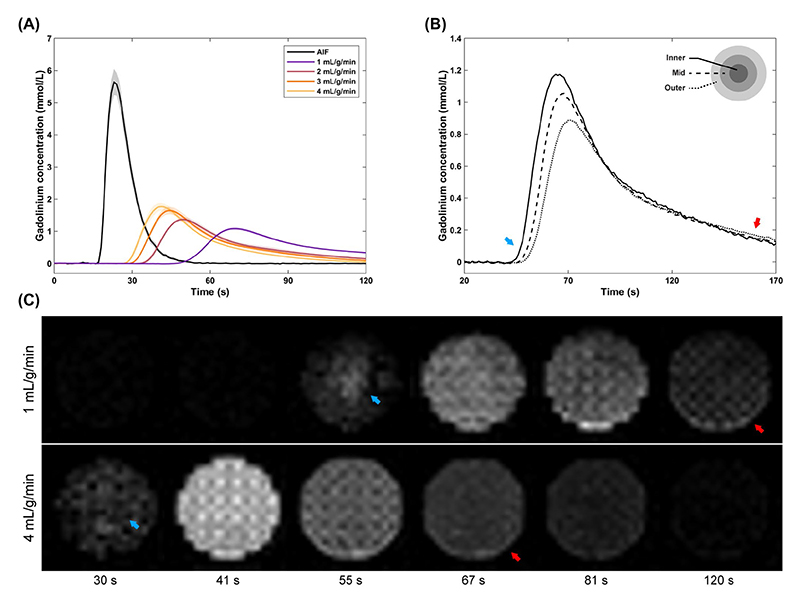
Dynamic contrast enhancement in the phantom. Plot (A) shows the mean arterial input function (AIF) sampled in the aorta and the mean myocardial tissue curves for four different reference mean perfusion rates. The shaded areas correspond to the standard deviation in five repeats. Plot (B) shows the myocardial enhancement in three transmural layers for an example scan at 1 mL/g/min mean perfusion rate. Corresponding myocardial images at six time points for 1 and 4 mL/g/min are also shown (C). Capillaries at the cross-sectional center of the myocardium demonstrate faster contrast uptake (blue arrows) and clearance (red arrows), whereas outer capillaries have a delayed uptake and clearance

Figure 5 shows the mean AIF and myocardial enhancement for all applied perfusion rates, the enhancement in three different myocardial transmural layers, as well as example DCE-MRI images demonstrating the contrast agent passsing through the myocardium. Higher applied flow rates lead to an increase in the contrast uptake and clearance rate in and out of the myocardium respectively. The measured gadolinium concentration is equivalent to values typically measured in clinical first-pass perfusion data (peak AIF concentration 5.65 ± 0.41 mmol/L). Perfusion images also demonstrate the spatial dependence in myocardium flow, with capillaries at the center undergoing faster uptake and clearance than outer capillaries (Figure 5). The mean baseline *T*_1_ measured in the aorta was 1896 ± 117 ms.

**Figure 6:**
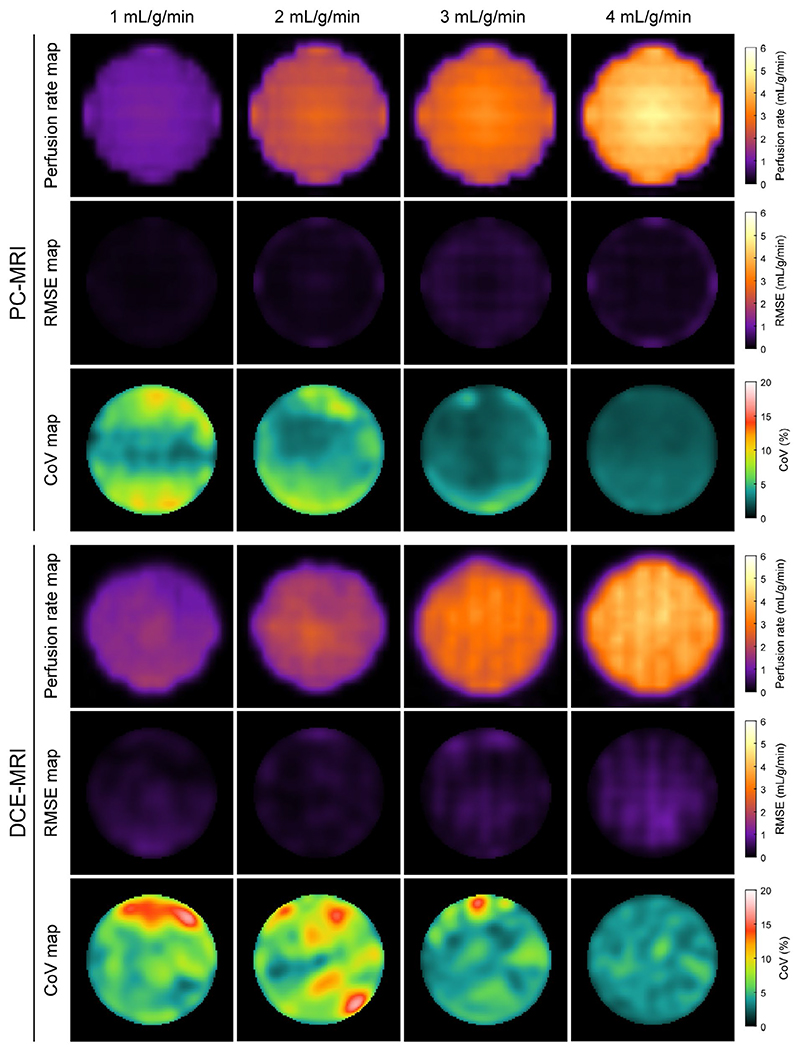
Pixel-wise assessment of perfusion rate by phase contrast (PC)-MRI and dynamic contrast-enhanced (DCE)-MRI. Perfusion rate maps and corresponding root mean square error (RMSE) and coefficient of variation (CoV) maps are shown, assessing the accuracy against reference maps and the repeatability respectively. The surrounding static tissue was cropped out of the maps to enhance visualization. The reference maps were generated using computational fluid dynamics and are provided in Supporting Information Figure S2

Figure 6 shows example perfusion rate maps and corresponding RMSE and CoV maps for PC-MRI and DCE-MRI. Mean perfusion rate was accurately estimated by both methods (mean RMSE ranged between 0.09 and 0.23 mL/g/min for PC-MRI, and between 0.11 and 0.25 mL/g/min for DCE-MRI; Table 1). The lowest accuracy was measured for reference 3 mL/g/min for PC-MRI and 1 mL/g/min for DCE-MRI. Perfusion rate was highly repeatable, with CoV estimates generally decreasing with increasing perfusion. CoV ranged between 2.4% and 3.5% for PC-MRI and between 3.7% and 6.2% for DCE-MRI. The short-term repeatability in PC-MRI-based perfusion, as measured across the 10 phase-difference images per scan, was higher (CoV 0.3-3.3%; Supporting Information Figure S3). Regional patterns of RMSE and CoV differed between the two methods; PC-MRI typically demonstrated a uniform variation from the center to the periphery of the myocardium, whereas a more complex pattern was observed for DCE-MRI (Figure 6).

**Figure 7:**
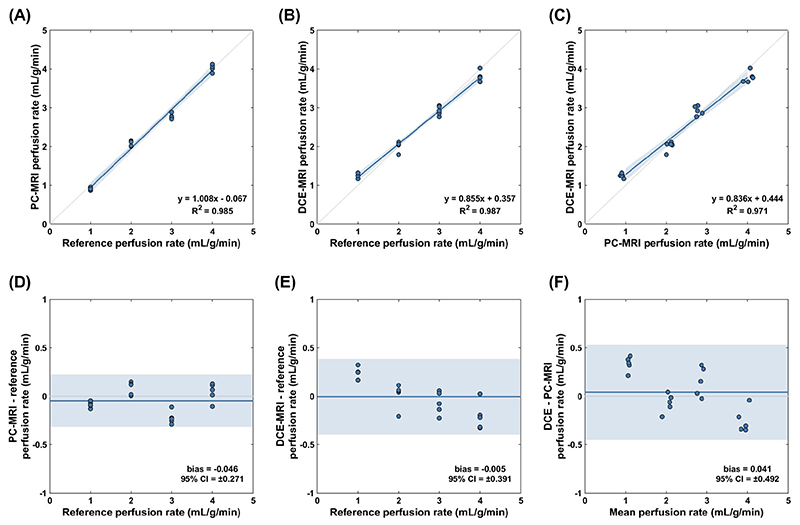
Linear regression and Bland-Altman analysis for mean perfusion rate. Measurements for phase contrast (PC)-MRI (A,D) and dynamic contrast-enhanced (DCE)-MRI (B,E) were compared against reference values provided by the system’s flow meters. Plots (C,F) show the correlation and agreement between the two MRI methods. The solid blue lines indicate the regression line in linear regression plots and the mean difference (bias) in perfusion rate in Bland-Altman plots. The shaded areas correspond to the 95% confidence intervals (CI). *P* < .001 for all linear regression coefficients

Mean perfusion rate had an excellent correlation with reference values for both PC-MRI and DCE-MRI (R^2^ = 0.985 and R^2^ = 0.987 respectively, both *P* < .001; Figure 7). Bland-Altman analysis suggested that both methods underestimate perfusion only marginally across the whole tested range (bias −0.05 and −0.01 mL/g/min for PC-MRI and DCE-MRI respectively). When compared together, the two methods had an excellent linear correlation (R^2^ = 0.971, *P* < .001) and a small bias of 0.04 mL/g/min. Analysis of variance did not uncover a significant difference in mean perfusion rate between methods and ground truth (*F*(1.442, 27.391) = 0.631, *P* = .490).

**Figure 8:**
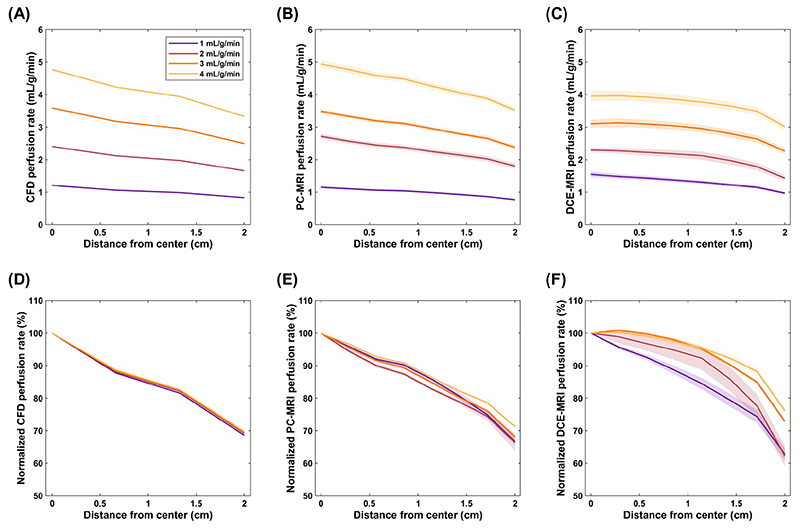
Transmural perfusion rate profiles in the synthetic myocardium. Plots show the perfusion rate averaged across line profiles sampled radially from the center to the periphery of the myocardial cross-section (mean of 100 random line profiles per map). Measurements were obtained in perfusion maps generated using computational fluid dynamics (CFD) (A), phase contrast (PC)-MRI (B) and dynamic contrast-enhanced (DCE)-MRI data (C), for four different reference mean perfusion rates. Plots (D-F) show the respective measurements normalized to the value in the center. The shaded areas in PC-MRI and DCE-MRI plots correspond to the standard deviation in repeats. Sharp changes across the profiles are due to partial volume effects arising from the finite size of the capillaries and the imaging resolution

Reference pixel-wise maps by CFD demonstrated a linear transmural variation across the full physiological range of applied perfusion rates, with a percentage difference between the myocardial center and periphery between 30.2 and 31.4% (Figure 8; maps in Supporting Information Figure S2). The transmural variation in PC-MRI maps largely matched the reference data (28.8-33.7%), whereas transmural DCE-MRI profiles were less linear and less reproducible for each applied perfusion rate (range in transmural variation 23.8-37.7%). However, the reproducibility was improved at higher perfusion rates for both MRI methods (Figure 8).

## 4 Discussion

In this study, a perfusion phantom generating spatially dependent flow values was developed to facilitate widespread utility as a true physical standard for pixel-wise myocardial perfusion. State-of-the-art quantification methods are currently assessed for accuracy against clinical reference methods, such as PET perfusion and invasive coronary angiography.^3,19,34^ However, none of these techniques constitute clearly defined and transferable gold standard methods for systematic and widespread validation, which limits the assessment of quantification reproducibility based on imaging with the same scanner.^35^ The performance of a physical standard is not confounded by physiological or pathological variability and allows direct validation and comparison of the same or different methods within or between systems. As such, it is the only approach that could enable universal standardization of methodologies.

The synthetic myocardium can be used in conjunction with a phantom setup providing reference perfusion rates for the assessment of clinical CMR first-pass perfusion protocols. Previously described perfusion phantoms offer only measurement of spatially homogeneous flow in less reproducible imaging setups.^20,22,26^ In contrast, the synthetic myocardium presented here consists of a capillary compartment mimicking the physiological diversity in capillary size and the transmural variation in perfusion between inner and outer myocardial layers. Pixel-wise perfusion quantification can be assessed based on its accuracy and reproducibility in depicting the transmural gradients, the extent and pattern of which is precisely known from the designed geometry and CFD simulations. Therefore, the proposed solution offers a more dedicated and versatile assessment of parameters pertaining to perfusion data acquisition and analysis and holds promise for use as a quality assurance tool in the clinic.

Reference myocardial flow rates are regulated using peristaltic pumps and monitored by ultrasonic flow meters calibrated at an uncertainty of ±1%. To allow cross-method assessment and validation, the reference flow rates can be converted to perfusion rates based on the principles of the indicator-dilution theory.^28^ Perfusion rates estimated using PC-MRI were found to have an excellent correlation and agreement with applied values at the global and pixel-wise level. The coefficient of variation improved for higher perfusion rates and toward the central capillaries of the myocardium. However, this may not reflect the repeatability of the phantom itself because the SNR of PC-MRI increases as myocardial velocities approach the used VENC value and the method becomes more reliable. Low VENC values are required for lower myocardial flow rates but may not be achievable by most clinical scanners due to requirements of large flow-encoding gradients. The short-term repeatability in perfusion rate measured across phase-difference images in individual PC-MRI scans was higher than the long-term repeatability measured across repeated scans. The variability across scans may have been overestimated by the data analysis process, which involved estimation of scan-specific but not image-specific eddy current-induced velocity offsets. Nevertheless, the stability of the phantom is sufficiently high to enable routine use in short- or long-term imaging experiments. Furthermore, the excellent correlation of PC-MRI-based mean perfusion rate with reference values and the low pixel-wise errors suggest that the method is well suited for the calibration and verification of the phantom’s performance.

The myocardium’s transmural variation in perfusion was optimized and calibrated by CFD simulations. Measurements by PC-MRI matched the expected transmural variation of 30% and had an excellent repeatability for all applied mean perfusion rates. The transmurality measured with DCE-MRI was less repeatable and deviated from linearity. This is primarily a consequence of the vastly more complicated DCE-MRI ac-quisition and analysis process compared with PC-MRI. First, perfusion quantification is based on measuring the myocardial signal enhancement during the first pass of the contrast agent and assumes that the SI is linearly proportional to gadolinium concentration. However, signal saturation is a well-known pitfall in CMR perfusion and could have affected not only the very high AIF signal but also myocardial SI toward higher perfusion rates, explaining the corresponding decrease in measured perfusion rate. A dual-sequence technique was used to account for saturation effects, and SI was converted to gadolinium concentration using a well-established signal model.^31^ However, SI conversion can only partially restore linearity and improve quantification accuracy and at the cost of added complexity to the imaging protocol.^2,36^ Second, pixel-wise quantification by curve fitting inherently filters the data and may have smoothed out the observed transmural variability in perfusion. A previous study using four different deconvolution models to analyze phantom data hinted that the pixel-wise pattern of perfusion largely depends on the model used.^5^ Fermi function-constrained deconvolution was used in this study as it is widely employed, is robust to noise, and has an excellent diagnostic performance in patients compared to other common approaches.^5,27,37^ An alternative model could have improved the accuracy of perfusion estimates but less likely the quality of pixel-wise maps. Lastly, partial volume effects arising due to the finite size of the myocardial capillaries and the imaging resolution may have contributed further to the variability in transmural measurements. The finite size of the capillaries also explains the small difference from the expected 30% transmural variation in simulated maps, but this was minimal compared to differences in MRI data and did not have a significant impact on pixel-wise comparisons.

The issues described here constitute just a small body of evidence supporting the need for a physical standard for CMR perfusion. The wide variety of available imaging methods undermines the reliability of this clinical tool and prohibits effective pooling of data in multicenter studies. A strategy to ensure that measurements from each scanner are accurate and repeatable is therefore essential. The phantom can be used for optimization and validation of each aspect of the imaging pipeline from acquisition to quantification and can potentially be integrated into any CMR facility’s quality assurance protocol.

### 4.1 Limitations

An attempt to develop a synthetic myocardial tissue as a physical standard expectedly comes with important limitations. The myocardium is a single-compartment tissue model and, as such, it lacks true physiology. Common extravascular MRI contrast agents diffuse in the human myocardial tissue in a considerably more complex way that involves–in its simplest form–prolonged contrast exchange between the vascular and extravascular spaces before its total clearance.^38,39^ Therefore, the proposed phantom is more suited for perfusion models describing intravascular contrast dynamics but could potentially be used for the validation of multicompartmental tracer kinetic models under the assumption that no extravasation takes place. Additionally, designing a phantom with multiple compartments is markedly more challenging and could possess further technical and practical considerations with regard to control and monitoring of additional parameters, such as the contrast exchange rate. A further limitation of this study is that the transport liquid used is water instead of blood. Water has a different *T*_1_ value than blood as well as lower density and viscosity. We used gadolinium-doped water to ensure baseline *T*_1_ values similar to blood are measured, but an impact on the apparent MRI signal and contrast agent flow dynamics may have still been present. Regrettably, the use of blood or blood-mimicking liquids in a phantom setup requiring constant supply for an extended period of time is not only costly but can significantly reduce the shelf life of the various system components.

## 5 Conclusions

A novel 3D printed synthetic myocardial phantom simulating transmural myocardial perfusion gradients was developed. The phantom generates realistic enhancement curves across the full physiological range of perfusion rate and was found to be accurate and repeatable using PC-MRI and clinical DCE-MRI. The phantom can be used for effective pixel-wise assessment and validation of CMR first-pass perfusion methods and can become an essential tool for quality assurance in the clinic. This can ultimately help reduce the number of animals and patients that are essential for current validation methods.

## Supplementary Material

Supporting information

## Figures and Tables

**Table 1:** Mean myocardial perfusion rate and metrics of accuracy and reproducibility for PC-MRI and DCE-MRI

	PC-MRI			DCE-MRI		
Reference perfusion rate (mL/g/min)	Estimated mean perfusion rate (mL/g/min)	RMSE^a^ (mL/g/min)	CoV (%)	Estimated mean perfusion rate (mL/g/min)	RMSE (mL/g/min)	CoV (%)
1.00	0.91 ± 0.03	0.09	3.5	1.25 ± 0.06	0.25	4.4
2.00	2.08 ± 0.07	0.10	3.2	2.01 ± 0.13	0.11	6.2
3.00	2.78 ± 0.07	0.23	2.4	2.93 ± 0.12	0.13	4.0
4.00	4.04 ± 0.10	0.10	2.4	3.79 ± 0.14	0.24	3.7

a Root mean square error (RMSE) and coefficient of variation (CoV) measurements were obtained based on mean perfusion rates and may differ from apparent regional values in the pixel-wise maps in Figure 6. DCE-MRI, dynamic contrast-enhanced MRI; PC-MRI, phase contrast MRI.
