# Supplementary material for: Pixel-wise assessment of cardiovascular magnetic resonance first-pass perfusion using a cardiac phantom mimicking transmural myocardial perfusion gradients
Source: Magn Reson Med. Author manuscript; Available in PMC 2021 Jul 12. (PMC7611223; doi:10.1002/mrm.28296)
Supplement: Supporting information [file EMS128760-supplement-Supporting_information.pdf]

Supporting Information

Assessment of flow behavior in myocardial capillaries

The velocity of a liquid flowing through a pipe decreases with increasing tube length when the total pressure difference between the outlets is kept constant. The extend of this decrease depends on whether the flow is laminar or turbulent; for laminar flow, the relative decrease in flow rate is inversely proportional to the relative increase in pipe length, whereas for turbulent flow, a more complex behavior is observed. The flow regime can be roughly determined by estimating the Reynolds number; if this number is smaller than a critical value, the flow is likely to be laminar, if it is larger than a different critical value, the flow is likely turbulent, and otherwise something in between. The Reynolds number is defined as

$$Re = \frac{\rho u l}{\mu} = \frac{u l}{\nu}$$

where ρ is the density of the fluid (kg/m^3), u is its velocity (m/s), μ is its dynamic viscosity (Pa·s or kg/m·s), and l is the characteristic linear dimension of the tube (m). The ratio μ/ρ is the kinematic viscosity ν (m^2/s). l is the dimension that defines the length scale of a physical system. For a circular or non-circular pipe, l can be its hydraulic diameter defined as $D_h = 4A/P$, where A is the pipe's cross-sectional area (m^2) and P is its wetted perimeter (m). Therefore,

$$Re = \frac{4Au}{P\nu} \quad (1)$$

For example, D_h is the inner diameter of a cylindrical pipe or the length of the side of a square duct. As follows from equation (1), the Reynolds number is small at low velocities or small hydraulic diameters, for which the flow regime is typically laminar. At high velocities, eddy currents cause flow turbulence. Generally, laminar flow occurs when $Re < 2300$ and turbulent flow when $Re > 4000$.

In the developed myocardial compartment, the capillaries have square and octagonal cross-sectional shapes and, therefore, we can estimate their Reynolds number using their hydraulic diameters. The Supporting Information Table S1 below lists A and P for all five different types of capillaries measured from the known geometry, as well as their D_h. The Reynolds number was estimated with equation (1) using the kinematic

viscosity of water at 25 °C, $\nu_w = 8.927 \cdot 10^{-7} \mathrm{m}^2/s$, and a maximum velocity $u_{max} = 2.5 \cdot 10^{-2} m/s$ measured by phase-contrast MRI and computational fluid dynamic simulations during prototype testing (see also Supporting Information Figure S2 showing simulated maps for the final product).

The Reynolds number for all capillaries is well below the critical value and therefore the flow regime is laminar. This means that we expect a transmural flow variation across the myocardial capillary compartment of the same extent as the variation in capillary length (30%).

Supporting Information Table S1. Flow characteristics of myocardial capillaries.

$A\ (\cdot 10^{-6} m^2)$	$P\ (\cdot 10^{-2} m)$	$D_h\ (\cdot 10^{-4} m)$	Re
6.37	9.26	2.75	77
3.20	7.16	1.79	50
1.93	5.56	1.39	39
1.43	4.78	1.20	34
1.00	4.00	1.00	28

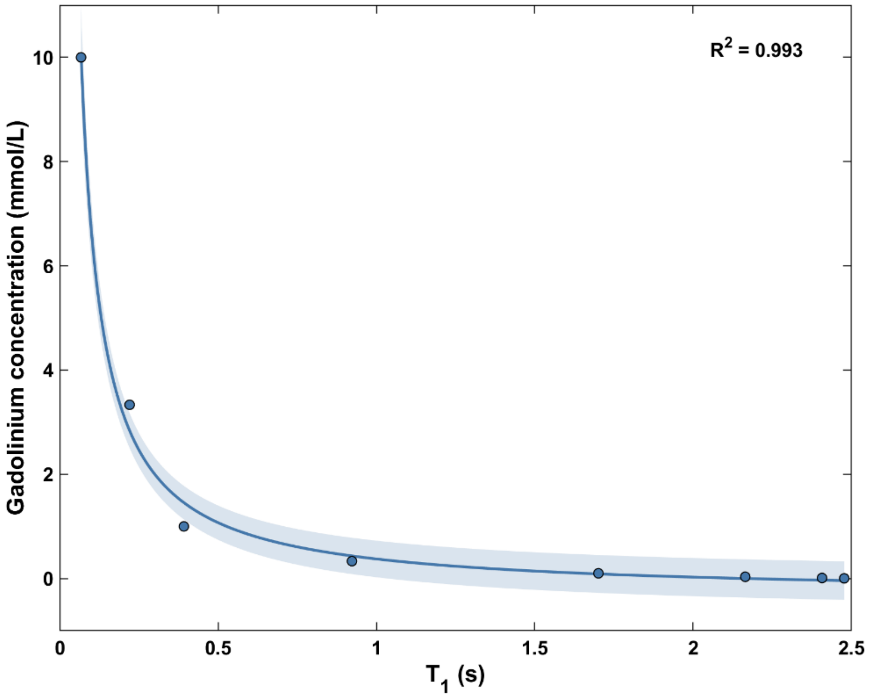

Supporting Information Figure S1. Gadolinium concentration versus T_1. Eight tubes with various concentrations of Gadobutrol were used between 0 and 10 mmol/L and T_1 was measured using the MOLLI sequence. Data fitting was performed using the formula $C = (1/r_1)\,(1/T_1 - 1/T_1(0))$, where r_1 is the relaxivity of the contrast agent and $T_1(0)$ is the baseline (pre-contrast) T_1; both were set as free parameters for fitting. The shaded areas correspond to the 95% confidence intervals of the regression line. Using the formula, a concentration of 0.1 mmol/L was estimated for a required T_1 = 1846 ± 109 ms (measured in a population of 30 patients and healthy volunteers scanned with the same sequence and on the same scanner).

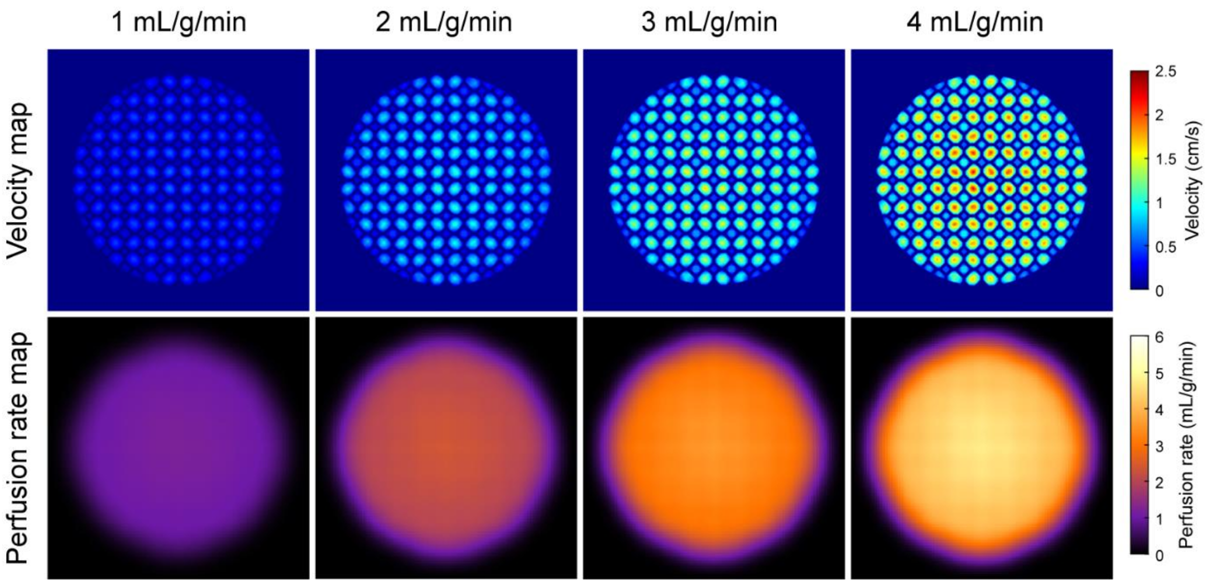

Supporting Information Figure S2. Reference velocity and perfusion rate maps. The maps were generated using computational fluid dynamic simulations for applied global flow rates between 100 and 400 mL/min (perfusion rate 1-4 mL/g/min). The velocity maps (top row) were converted to perfusion rate maps by multiplication with the known total capillary cross-sectional area (9.24 cm^2) and division by the dispersion volume (100 mL corresponding to 100 g of water; see Figure 4 of the main manuscript). The perfusion rate maps were scaled to the resolution of the MRI data, smoothed with a Wiener filter and scaled back to the original resolution (bottom row) for evaluating the accuracy of MRI data.

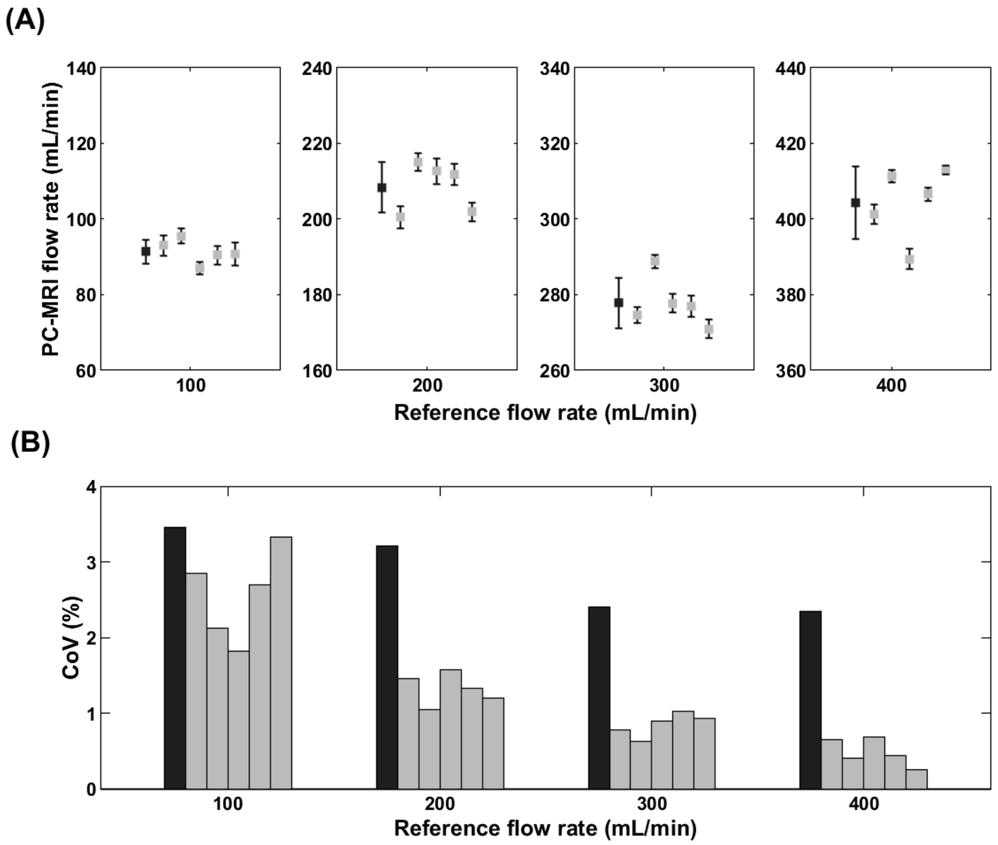

Supporting Information Figure S3. Flow rate measurements by PC-MRI. Plot (A) shows the mean myocardial flow rate measured across the 10 phase-difference images per repeated scan (gray squares), as well as the mean across the five scans (black squares). The error bars indicate the standard deviation (SD). Plot (B) shows the corresponding coefficient of variation (CoV). Successive phase-difference images were acquired with a 100 ms delay, thus the CoV for each scan (gray bars) indicates the short-term flow reproducibility in the myocardium. PC-MRI scans were acquired every 10 minutes on average, thus the CoV across scans (black bars) indicates the long-term flow reproducibility during a typical phantom experiment.
